# 支气管结核的外科治疗

**DOI:** 10.3779/j.issn.1009-3419.2018.04.19

**Published:** 2018-04-20

**Authors:** 军忠 阮, 天辉 张, 福根 李, 勇 段, 鸣 韩, 子彤 王

**Affiliations:** 101149 北京，首都医科大学附属北京胸科医院胸外科 Department of Thoracic Surgery, Beijing Chest Hospital, Capital Medical University, Beijing 101149, China

**Keywords:** 支气管结核, 支气管狭窄, 外科治疗, Endobronchial tuberculosis, Bronchial stricture, Surgical treatment

## Abstract

**背景与目的:**

支气管结核是肺结核常见的并发症，本研究目的是探讨支气管结核的临床特点，手术指征及总结外科治疗经验。

**方法:**

对36例诊断明确且造成支气管狭窄或肺不张的支气管结核患者，给予药物及内镜腔内治疗无效而进行外科治疗，术前规律抗结核治疗6个月以上，术后继续抗结核治疗9个月-12个月。其中全肺切除8例，肺叶切除23例，袖状肺叶切除5例。

**结果:**

36例患者均治愈，无手术死亡，6例患者术后出现并发症，给与对应治疗痊愈出院，无支气管胸膜瘘及结核播散。随访1年以上无复发。

**结论:**

对于药物及内镜治疗无效，伴有肺内不可逆病变的支气管结核患者应考虑积极手术。

## Abstract (en)

**Background and objective:**

Bronchial tuberculosis is a common complication of pulmonary tuberculosis. The present report is to investigate and analyze the indication and efficacy of surgical treatment of bronchial stricture due to severe endobronchial tuberculosis, when the drug and endoscopic treatment were no effect.

**Methods:**

Reviewed the clinical-pathological records documenting the surgical outcomes in 36 bronchial stricture due to severe endobronchial tuberculosis who underwent lobectomy or pneumonectomy enrolled in our hospital between January 2000 and February 2016. Pneumonectomy in 8 cases, lobectomy in 23 cases, sleeve resection in 5 cases.

**Results:**

No intraoperative or early postoperative death occurred. Six patients developed complications. All 6 cases recovered well after treatment.

**Conclusion:**

Surgical treatment is still the recommended treatment modatity for bronchial stricture caused by endobronchial tuberculosis due to its good results. It should be performed in time when the drug and intraluninal treatment were no effect for avoiding of being progeressed.

支气管结核是由于结核杆菌侵袭支气管黏膜及黏膜下层而造成的，即使给予充分的抗结核治疗，相当多的支气管结核导致的狭窄也会进一步加重。重度结核性支气管狭窄及闭锁常伴有阻塞性肺炎、支气管扩张症和毁损肺等严重的并发症和后遗症，治疗相当棘手，常需要外科手术。

我院自2000年1月-2016年2月，共收治36例支气管结核患者在充分抗结核药物治疗及支气管镜介入治疗不愈，给与外科手术治疗，现将这些患者的病史、手术方式、手术前后的治疗以及随访资料进行了回顾性分析，探讨支气管结核的外科治疗的适应证，手术方式及效果。

## 1 资料与方法

### 1.1 一般资料

本组36例，男性15例，女性21例，年龄16岁-55岁，平均年龄32.3岁。所有患者均经胸部计算机断层扫描（computed tomography, CT）及支气管镜检查，病理或涂片查到结核菌，证实为支气管结核，其中40岁以下者29例，占80.5%。临床表现：本组患者中，以咳嗽、咳黄痰为主的患者23例，占63.9%，伴有发热的患者7例，占19.4%；反复咳血或痰中带血者8例，占22.2%；胸闷气短11例，占30.6%；胸痛4例，占11.1%，部分患者伴随多个症状。

### 1.2 临床检查

全组均经胸部CT检查，其中以肺不张多见，占69.4%（25/36），全肺实变不张及毁损8例，肺叶不张17例；17例肺叶内有散在硬结或条索病变，其中6例与支气管结核病变部位不一致。所有患者术前均行支气管镜检查，见支气管皆有不同程度的狭窄，30例狭窄超过管腔的2/3，其中结核性支气管狭窄发生于右侧16例，其中右主支气管2例，上叶支气管7例，中间段支气管5例，下叶支气管2例；左侧20例，其中左主支气管6例，上叶支气管7例，下叶支气管7例。术前镜下检查支气管黏膜无充血水肿，术前痰菌阳性5例，其中耐药3例；痰菌阴性31例。

术前肺功能第1秒用力呼气量（forced expiratory volume in 1 s, FEV_1_）1.13 L-3.96 L，平均1.87 L。对拟行全肺切除FEV_1_ < 1.8 L，肺叶切除FEV_1_ < 1.5 L的患者行通气血流检查，剩余肺预测肺功能FEV_1_ > 1.0 L方行肺切除术, 所有患者均行血气分析检查，PaO_2_≥70 mmHg，PaCO_2_≤45 mmHg。

### 1.3 治疗

本组患者术前均经过内科正规抗结核治疗6个月以上。33例患者给与支气管镜腔内治疗，粘膜给药33例，球囊扩张20例，冷冻治疗13例，部分患者先后给与多种治疗，术后继续抗结核治疗9个月-12个月。全组患者36例，其中全肺切除术8例（其中左侧6例，右侧2例）；袖状肺叶切除术5例（其中左上叶3例，右上叶2例）；肺叶切除23例，其中单纯左上叶切除术4例，左下叶切除术7例，单纯右上叶切除5例，右下叶切除2例，复合肺叶右中下叶切除术5例。常规开胸手术28例，胸腔镜手术8例，其中因粘连严重中转开胸2例。

## 2 结果

36例患者均治愈，无手术死亡，无支气管胸膜瘘及结核播散。术后出现并发症6例（16.7%），其中肺部感染2例，术后残腔2例，脓胸1例、切口感染l例，经对症及对因治疗均痊愈。患者经病理学检查均证实为支气管结核，15例伴肺内结核。术后所有患者继续抗结核治疗9个月-12个月，随访12个月，未发现复发。

## 3 讨论

支气管结核是指由于结核杆菌侵袭支气管黏膜、黏膜下层或进一步破坏肌层及软骨，最终瘢痕愈合，导致支气管狭窄或完全闭塞的一种疾病，是肺结核的一种特殊类型。支气管结核是肺结核常见的并发症，报道显示有约10%-40%的活动性肺结核患者合并有支气管内膜结核^[1]^，此类疾病单纯全身用药治疗时间长，部分病例达1年以上的治疗，且遗留不同程度的并发症。支气管内膜结核的发病机制尚未完全明确，可能的机制包括：邻近肺实质病变的直接侵犯，痰中所带结核菌在气道中种植，血行播散，淋巴结侵蚀支气管以及沿支气管树的淋巴播散^[2]^。支气管结核好发于左、右主支气管，两肺上叶、下叶支气管。并且常为多部位受累，多种病变并存，本组患者术前支气管镜检查，可见支气管皆有不同程度的狭窄，30例狭窄超过管腔的2/3，占83.3%。大多数报道提示女性支气管内膜结核发病率高于男性，而且多发于20岁-30岁，其原因尚不清楚^[3, 4]^，本组36例患者中，女性21例，占58.3%，与文献报道相一致。支气管结核在临床上易被误诊或漏诊，可以导致支气管狭窄、肺不张甚至毁损肺等严重并发症，而且支气管结核也是结核病的一个重要传染源，所以早期诊断十分重要。本组患者表现以咳嗽、咳黄痰多见，占63.9%；另外可伴有发热、胸闷气短、咯血或痰血等，易被误诊为常见肺部疾病。目前诊断支气管结核的手段多为痰结核菌检查、影像学检查、支气管镜检查等。胸部CT检查可以显示胸部不变，例如纵隔淋巴结肿大，肺部空洞以及胸膜病变，还可以显示支气管腔是否狭窄，胸部CT的3D气道重建可以评估气道狭窄程度、长度，甚至有助于后续内镜介入及外科治疗^[5]^，但目前认为支气管镜检查是支气管结核病最具诊断价值的方法。支气管结核患者大多可通过支气管镜刷检及活检明确诊断^[6]^，17%-79%的支气管结核患者可以通过支气管镜检查确诊^[7]^。本组患者术前均行气管镜检查，镜下检查支气管黏膜无充血水肿，术前痰菌阳性5例，其中耐药3例；痰菌阴性31例，本组患者支气管镜活检及刷检阳性率较低，仅为13.9%（5/36），其主要原因是大多数内膜结核已给予药物治疗并处于瘢痕愈合期，支气管无活动性结核，考虑与术前至少6个月的抗结核治疗有关，这与Watanabe等^[8]^的报道一致。

根据支气管镜所见，支气管结核可分为以下7种镜下病变亚型^[6]^：活动干酪型、水肿充血型、纤维狭窄型、类肿瘤型、肉芽肿型、溃疡型以及非特异性支气管炎症型，不同的亚型病变可以共存，也可以转化进展为另外一个亚型病变，水肿充血型、纤维狭窄型、类肿瘤型三个亚型在没有接受正规治疗，在3个月内大部分病变最终进展为支气管狭窄或闭锁^[6]^。部分学者认为应该采用球囊扩张或者支架植入的方法恢复管腔的通畅。但是长期的观察显示球囊扩张只能收到暂时性的疗效，常需要反复扩张^[9]^。支架的长期效果也不确定而且作为异物又可以造成肉芽组织增生从而导致支气管狭窄，本组36例患者中，33例患者给与支气管镜腔内治疗，腔内粘膜给药33例，球囊扩张20例，冷冻治疗13例，部分患者先后给与多种治疗，但效果欠佳，所以对于支气管严重瘢痕狭窄以及伴随后遗症患者，气管镜腔内介入治疗往往无效，需外科手术。

支气管结核外科手术治疗的时机和方式：一般手术时机应选择在患者经过充分、规范的抗结核治疗，支气管结核处于非活动期，而且经内镜介入治疗无效^[10]^。本组患者均经过内科正规抗结核治疗6个月以上，且术前支气管镜检查证实病变处于稳定期。手术方式的选择应根据病变部位、累及范围、狭窄的严重程度及远端肺组织的实际情况（如肺结核的病灶范围和肺毁损程度等）以及患者的肺功能状况而定。多数学者认为袖式肺叶切除是治疗结核性支气管狭窄的首选手术方式。但笔者认为，支气管结核手术方式的选择应根据病变部位、累及范围、狭窄的严重程度、远端肺组织的病变程度、余肺情况以及患者的肺功能、机体状况而定。对于肺结核范围并发支气管结核部位一致未累及主支气管的患者，手术方式多采用将狭窄段支气管连同受累肺叶一并切除；而对于合并远端肺毁损、严重支气管扩张或肺不张等不可逆病变累及主支气管的患者，手术范围应包括狭窄的支气管及远端肺组织，术式多采用袖状切除术。本组袖状肺叶切除5例，术后未出现吻合口瘘及术后狭窄，收到较好效果。总之，支气管结核手术原则是既彻底切除结核病灶，又能最大限度地保留肺功能。对于支气管结核术后并发症的发生与肺结核手术大致相当，肺部感染与术后脓胸，切口感染与常规肺切除处理方法相同，但从理论上来说支气管结核患者进行肺切除手术支气管残端瘘风险较大，而支气管残端结核阳性是肺切除术后支气管残端瘘的高危因素^[11]^。支气管残端瘘是肺切除术后的严重并发症，死亡率可达40%以上，支气管断端局部炎症是其发生的危险因素。手术成功的关键在于切除瘢痕组织，在正常支气管黏膜上施行缝合，避免支气管残端内膜结核残留是预防支气管残端瘘的一个重要措施，结合本组手术患者效果，病人术后未出现支气管残端瘘或吻合口瘘，笔者考虑为防止术后此类并发症的发生应做到以下3点：①术前充分准备：应进行规范抗痨治疗，同时术前有效控制患者基础疾病（如糖尿病，高血压等），提高病人营养状态，术前支气管镜检查是必要的，镜下见手术侧支气管结核病灶不活动；②术中应保证足够的支气管切除长度、保证吻合口或残端无结核活动，细致的吻合或缝合，或应用直线缝合器缝合支气管残端，如果可能使用自体组织覆盖加固吻合口或残端等；③术后患者继续规律足量抗结核治疗9个月-12个月。这样可降低术后吻合口瘘的发生。

综上所述，外科手术是治疗支气管结核的重要手段，疗效满意。对药物治疗及介入治疗不愈的支气管结核，在给予充分抗结核治疗的基础上，严格评价病人术前情况，把握手术适应证条件，恰当的选择手术切除方式及切除范围，外科手术治疗能有效降低并发症及结核播散的发生，是一种安全有效的方法。
